# Effects of Chloroform on Uterine Contractions

**Published:** 1857-04

**Authors:** 


### *Effects of Chloroform on Uterine Contractions.*

In the January number of the North American *Medico-Chirurgical Review*, we find some very interesting remarks from the pen of Prof. John Hardin on the subject of "Transverse Presentations of the Fœtus." The following, however, has attracted our especial attention, both on account of its importance and its being altogether contrary to our own experience. While treating of remedies to be resorted to for the purpose of relaxing the uterus before undertaking version, the author says—"Fortunately, modern investigation has brought to our knowledge a remedy, prompt, efficient, and so nearly safe that we do not hesitate to employ it it in preference to all others. This remedy, I need scarcely say, is chloroform, used to the extent of producing as full and complete anæthesia as would be required for amputating a limb. It holds the uteriue action in complete abeyance, so that we may calmly and considerately employ any mode of changing the presentation of the child that we may desire."

Now, we are fully aware that the author does not stand alone in thus believing in the power that chloroform possesses over the contractility of the uterine muscular fibre. Even Simpson himself, the father of "chloroform in midwifery," speaks of the deep state of anæthesia suspending uterine action, or even causing relaxation of the organ. If it be true, that the organ can be made thus safely to relax its hold on the fœtus while we perform version, either cephalic or podalic, then are we surely possessed of a remedy which robs "cross births" of half their terror. Whether all this is true or not we will not now say, preferring to await a little more experience on the subject. Thus far, however (and we claim *some* experience in such matters,) we have never met with a case wherein even the most complete state of aæthesia has done more than delay the *recurrence* of the *expulsive* efforts of the uterus: whenever our hand has been introduced under such circumstances, we have found the normal tonic contractions of the organ on the child altogether unaffected. In other words, our own observation teaches us, that under the state of complete anæthesia the womb is closely applied to the ovoid form of the fœtus, and that any effort to perform version arouses the organ as fully as in a case where no chloroform has been administered.

[*N. O. Med. News & Gaz.*

